# Navigating Diagnostic Challenges: Primary Pulmonary Choriocarcinoma Depicted by Fluorodeoxyglucose (FDG)-Positron Emission Tomography-Computed Tomography (PET/CT)

**DOI:** 10.7759/cureus.69866

**Published:** 2024-09-21

**Authors:** Fatimah Abu Aljaaz, Serin Moghrabi, Dhuha Al-Adhami, Marwa Al-Shatti, Akram Al-Ibraheem

**Affiliations:** 1 Department of Nuclear Medicine and PET/CT, King Hussein Cancer Center (KHCC), Amman, JOR; 2 Department of Pathology, King Hussein Cancer Center (KHCC), Amman, JOR

**Keywords:** fdg, peripheral hypermetabolism, pet/ct, primary pulmonary choriocarcinoma, pulmonary cancer, renal metastasis

## Abstract

This report explores a noteworthy case diagnosed with primary pulmonary choriocarcinoma (PPC), a rare and often fatal non-seminomatous germ cell tumor. Initially misdiagnosed as lung adenocarcinoma, this case underscores the diagnostic complexities associated with PPC. A 44-year-old woman initially misdiagnosed with non-small lung cancer underwent unsuccessful chemoradiation. Emergency presentation with gastrointestinal bleeding revealed intestinal intussusception and severe anemia, rendering her ineligible for surgery. A thorough assessment, including fluorodeoxyglucose (FDG) positron emission tomography/computed tomography (PET/CT), uncovered an aggressive neoplastic pattern. A subsequent biopsy confirmed PPC. Ten cycles of chemotherapy (cisplatin and etoposide on day 1, followed by etoposide, methotrexate, and dactinomycin on day 8) were offered. A complete metastatic response and a marked primary tumor reduction were evident at the end of therapy by PET/CT. Our findings underscore the importance of whole-body FDG PET/CT in comprehensively evaluating disease extent, displaying aggressive and atypical patterns, and offering guidance in complicated cases under multidisciplinary settings.

## Introduction

Primary pulmonary choriocarcinoma (PPC) is a rare type of lung malignancy affecting both sexes [1]. It is characterized by a poor therapy response and unfavorable survival outcome [1]. The scarcity of cases has indeed made it challenging to identify the specific radiologic and molecular imaging features of PPC [2]. This rarity contributes to the often-delayed diagnosis of PPC due to its nonspecific presentation and the lack of awareness among clinicians. PPC constitutes a very small fraction of lung malignancies [2]. The exact percentage is difficult to pinpoint due to the extreme rarity of the condition [3]. To date, there is only a limited number of studies arguing PPC clinical characteristics and prognosis due to its rarity [3]. The overall prognosis of PPC is poor, with a 5-year survival rate below 5% [2]. This grim outlook is attributed to the aggressiveness of PPC, delayed diagnosis, and misdiagnosis, which often results in poor management and outcomes [3].

PPC development can be attributed to several hypotheses, including metastasis from gonadal choriocarcinoma, migration of retained primordial germ cells during embryogenesis, trophoblastic embolus from a gestational pregnancy after a prolonged latent period, or trans-differentiation of primary lung cancer into choriocarcinoma, with less than 80 known cases reported to date [3].

In this challenging case, we discuss the clinical course of a PPC patient whose initial disease was misdiagnosed as a case of lung adenocarcinoma. After unnecessary chemoradiation, fluorodeoxyglucose (FDG) positron emission tomography/computed tomography (PET/CT) was offered and identified as having an atypical neoplastic nature, necessitating histopathologic re-examination, which resulted in a definitive PPC diagnosis, effective management, and favorable response.

## Case presentation

**Figure 1:**
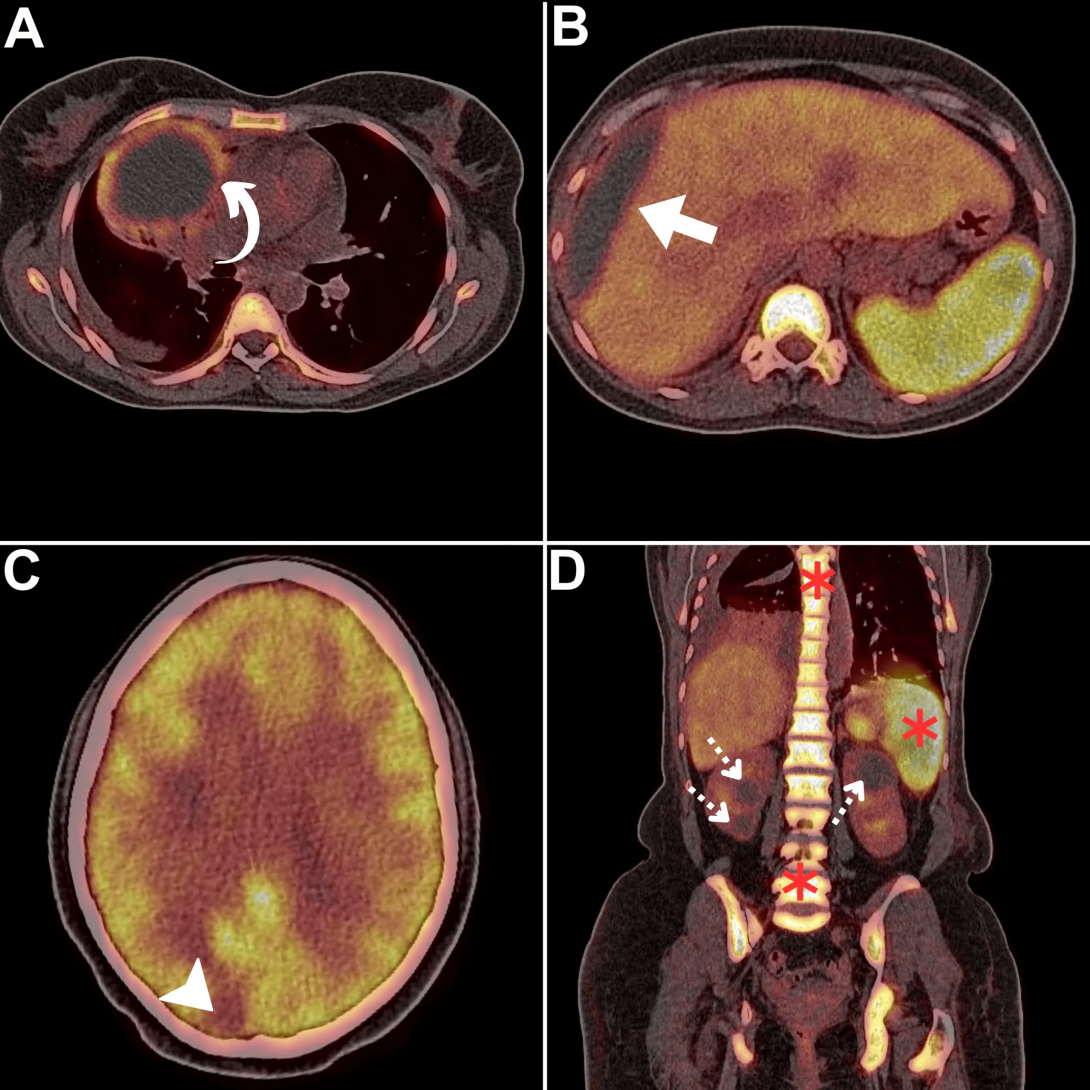
Baseline FDG PET/CT (A) Axial lung PET/CT revealed a single, large, peripherally hypermetabolic right upper lung lobe mass (curved arrow). (B, C) Axial PET/CT views demonstrated a large, hypometabolic occupying the lateral hepatic border (arrow) and brain (arrowhead). (D) Coronal PET/CT image exposed bilateral renal hypometabolic hypodensities (dotted arrows). Notably, intense bone marrow and splenic hypermetabolism was depicted (asterisks) correlating to ongoing anemia. FDG: fluorodeoxyglucose, PET/CT, positron emission tomography/computed tomography

A 44-year-old married woman, previously healthy, had a molar pregnancy three years ago. After terminating the molar pregnancy, she underwent a year of serial beta-human chorionic gonadotropin (β-HCG) surveillance. For two subsequent years, she remained asymptomatic. However, the emergence of amenorrhea and focal neurologic signs (headache, and confusion) prompted further consultation. Initial imaging reported a large right lung mass after undergoing a comprehensive evaluation through pan-computed tomography (CT) at an outside facility. She was misdiagnosed with lung adenocarcinoma based on lung biopsy. During this time frame, β-HCG and PET/CT were not performed. Chemoradiation therapy was offered for three months. The patient did not indicate any clinical improvement and was unable to recall additional details regarding the initially proposed treatment protocol. Persistent nausea and vomiting led to frequent emergency visits. Subsequently, she was transferred to our cancer center due to progressive upper gastrointestinal bleeding. Upon admission, she reported a negative family and psychosocial history. Further evaluation revealed intestinal intussusception, and due to severe anemia, surgery was deemed ineligible. A comprehensive biochemical and imaging assessment was promptly mandated. An FDG PET/CT scan was performed as part of the initial diagnostic workup (Figure 1).

**Figure 2:**
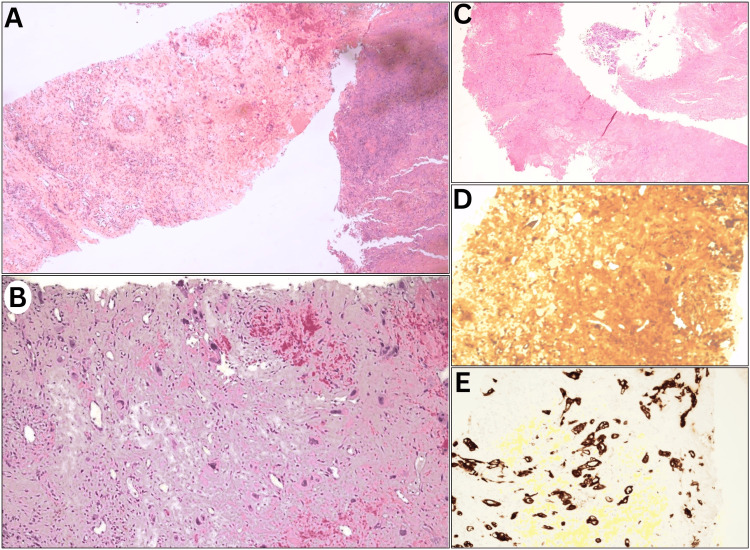
Histopathologic examination Histopathologic analysis of a redo lung biopsy confirmed primary pulmonary choriocarcinoma with extensive necrosis. (A-C) Hematoxylin and eosin stain exposed multinucleated pleomorphic cells, which presented alongside a large necrotic component. (D, E) Immunohistochemistry showed that choriocarcinoma cells were positively stained for (D) β-hCG and (E) CKMNF immunostains.

The scan revealed a picture of peripherally hypermetabolic right upper lung lobe mass (Figure 1A) and hypometabolic hypodensities within the lateral hepatic border, right cerebrum, and both kidneys (Figures 1B-1D). Such patterns suggest a unique, extensive, and aggressive neoplastic pattern that is atypical of lung adenocarcinoma based on radiologic and metabolic perspectives. Additionally, the assessment of hematologic, renal, and hepatic profiles was unremarkable except for anemia (hemoglobin of 8.7 g/dL), and markedly elevated levels of β-HCG were detected (135,000 U/L), necessitating a redo biopsy to rule out atypical metastatic or synchronous patterns. The second lung biopsy revealed features consistent with PPC, denoting extensive necrosis (Figure 2). Overall, Stage IV (T4N0M1c) PPC was confirmed.

**Figure 3:**
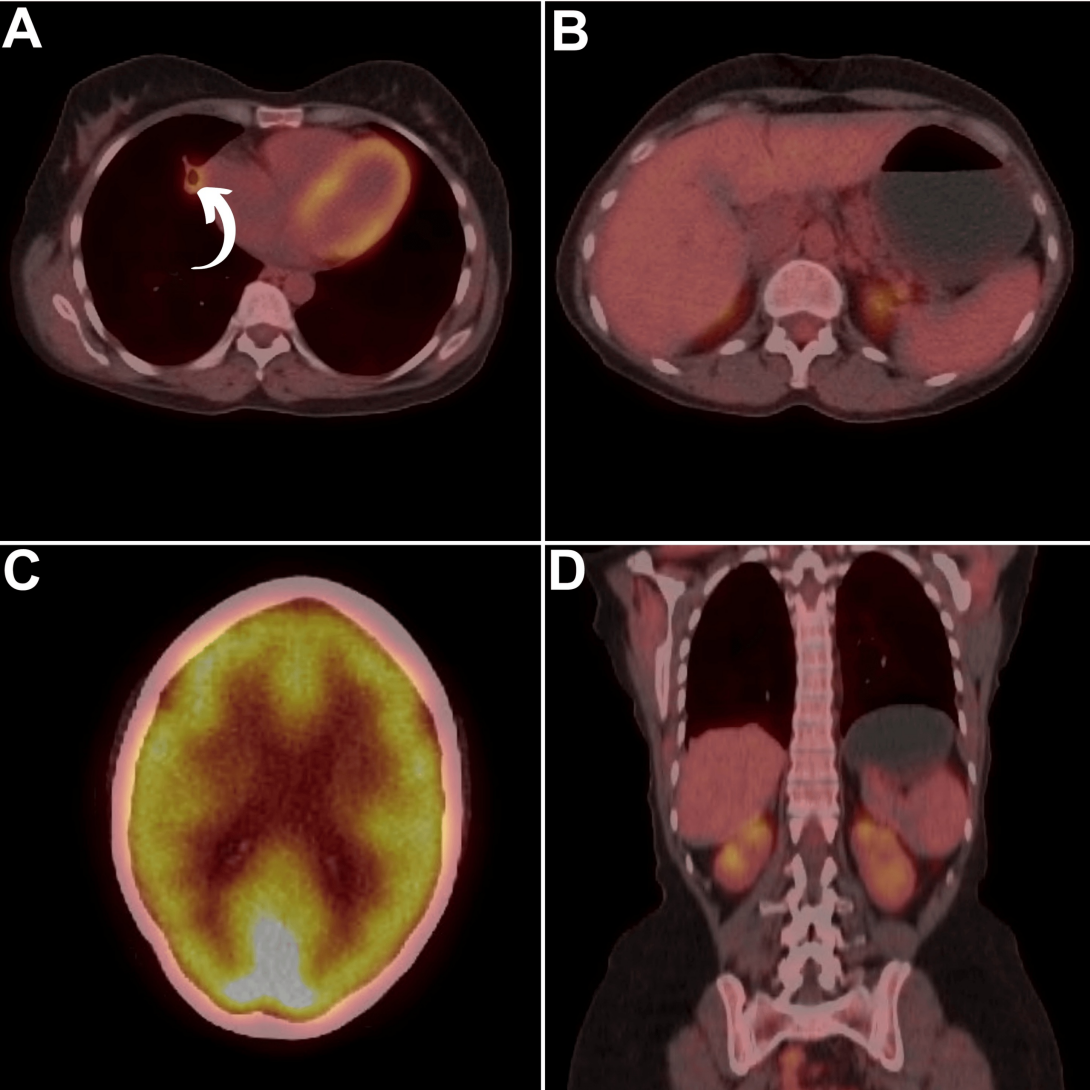
Follow-up fluorodeoxyglucose PET/CT (A) axial lung PET/CT, (B) axial hepatic PET/CT, (C) axial brain PET/CT, and (D) coronal PET/CT revealed evidence of complete metastatic response and a marked primary tumor reduction (curved arrow). PET/CT, positron emission tomography/computed tomography

A multidisciplinary evaluation recommended chemotherapy (cisplatin and etoposide on day 1, followed by etoposide, methotrexate, and dactinomycin on day 8). After 10 cycles, a complete metastatic response and a marked primary tumor reduction were evident on FDG PET/CT (Figure 3), accompanied by normalized β-HCG levels (1.1 U/L).

The patient is currently stable and undergoing surveillance for the coming months, with scheduled serial monitoring of β-HCG.

## Discussion

In most cases, PPC presents with several non-specific respiratory symptoms, including cough, chest pain, dyspnea, and hemoptysis [2-5]. In contrast, in our case, the cause that brought the patient was focal neurological signs misattributed to cerebral metastasis from lung adenocarcinoma, and she was therefore mistreated as such. In general, PPC poses a diagnostic challenge because of its non-specific and various signs and symptoms, in addition to overlapping radiological and cytological features [6-8]. Therefore, misdiagnosis can occur and has been reported previously in a 67-year-old man who was initially misdiagnosed as a case of non-small cell lung cancer via cytology when in fact he had PPC [1]. For PPC, early, accurate diagnosis and treatment are vital, as they can improve overall survival [2].

Typically, β-HCG levels are used as a tumor marker for choriocarcinoma, and elevated levels can be indicative of disease activity [9]. In the context of PPC, β-HCG levels can be significantly elevated, which helps in the diagnosis of this rare type of cancer. This was supported by our findings of excessive β-HCG levels initially, which were way higher than usual initial b-HCG levels for uterine choriocarcinoma, endorsing the Ikura hypothetical concept [10].

Few documented cases in the literature describe the FDG PET/CT pattern of primary and metastases in PPC. Primary PPC lesions might display variable degrees of FDG expression, as previous studies have indicated [11-14]. In our case, the primary PPC lesion exhibited peripheral hypermetabolism, indicating peripheral viability, contrasting with central necrosis, as supported by histopathologic analysis. Furthermore, metastatic lesions exhibited hypometabolism, a feature linked to ongoing necrosis, peripheral edema, or a cystic metastatic nature [15,14]. FDG PET/CT was a pivotal imaging tool to suspect the diagnosis, localize different metastases, guide a contributive biopsy, exclude the primary intrauterine choriocarcinoma, and assess the response to treatment.

With regards to metastatic patterns, PPC primarily spread homogenously, consistent with previous reports of lung, and brain involvement [3]. To our knowledge, this case marked the first observation of hepatic and renal metastasis from PPC.

## Conclusions

In this reported case, the utilization of FDG PET/CT proved essential in the detection and monitoring of PPC. This imaging modality is instrumental in delineating the full extent of the disease, particularly when it manifests in aggressive and non-conventional patterns. Furthermore, the incorporation of FDG PET/CT demonstrated considerable efficacy and reliability in evaluating therapeutic responses, thereby highlighting new potential avenues for the utilization of this hybrid imaging modality. In the context of a multidisciplinary approach, FDG PET/CT is invaluable, providing critical insights that inform the management of complex cases. The presence of atypical imaging findings on FDG PET/CT necessitates a thorough investigation, including histopathological examination, to ensure accurate diagnosis and the initiation of appropriate, timely therapeutic interventions. This comprehensive strategy is essential for optimizing patient outcomes in the face of this challenging and often unpredictable malignancy.
